# The ICF Classification as a Simple Tool to Aid in the Assessment of Healthcare Services in a Non-COVID-19 Hospital during the COVID-19 Pandemic

**DOI:** 10.3390/healthcare9040398

**Published:** 2021-04-01

**Authors:** Mateusz Lucki, Agnieszka Wareńczak, Ewa Chlebuś, Przemysław Daroszewski, Przemysław Lisiński

**Affiliations:** 1 Department of Rehabilitation and Physiotherapy, University of Medical Sciences, 60-545 Poznań, Poland; agnieszka.warenczak@gmail.com (A.W.); ewachlebus@ump.edu.pl (E.C.); plisinski@vp.pl (P.L.); 2 Department of Organization and Management in Healthcare, Poznań University of Medical Sciences, 60-545 Poznań, Poland; dyrektor@orsk.pl

**Keywords:** COVID-19, ICF, healthcare services

## Abstract

The COVID-19 pandemic has had a significant impact on the operation of medical facilities. In this period, they have seen increased absence of medical staff from work, a decrease in the number of hospitalizations and in the value of healthcare services provided. We assess the impact of this pandemic on the operation of a non-COVID-19 orthopedic and rehabilitation hospital using International Classification of Functioning, Disability and Health (ICF) categories. The authors analyzed these parameters in relation to the operation of a non-COVID-19 orthopedic, rehabilitation and rheumatological hospital in Q1 2020 compared to Q1 2019. For the analysis, the categories and qualifiers of the ICF were used, allowing for a simple and easily readable data analysis. In March 2020, in comparison to March 2019, the average working time of medical workers (*p* < 0.001) and the number of hospitalizations (*p* < 0.034) decreased significantly. In April 2020, compared to April 2019, the average working time of medical workers (<0.001) and the number of hospitalizations (0.002) also decreased significantly. In addition, in April 2020, the percentage value of the contracted services provided decreased significantly (*p* = 0.017), which was not observed in March of that year. The COVID-19 pandemic has affected the operation of a non-COVID-19 hospital, causing an increase in staff absences from work, a decrease in the number of hospitalizations and a decrease in the value of the revenue generated from health services provided. The ICF is a useful tool for the evaluation of a hospital’s healthcare services.

## 1. Introduction

The continuous spread of the COVID-19 pandemic has had a significant impact on private and professional life, including the operation of medical facilities [1]. Most facilities, especially hospitals, have been forced to reduce or alter the nature of the medical services they provide. Some hospitals have been dedicated solely to the care of patients with SARS-CoV-2 infection (COVID-19 hospitals) [2]. The medical staff working in these hospitals experienced increased burnout due to uncertainty about the future of the facility, tension and work overload, as well as staff shortages [3,4,5]. Previous studies have identified the factors contributing to burnout of healthcare workers, such as stress at work, anxiety and depression, which result from the direct nature of the work and the possibility of transmitting the infection to family members and the risk of social isolation [6,7,8]. The increasing rate of infection among medical staff resulted in increased absence from work [9,10]. In areas where the incidence of COVID-19 was particularly high, absences rose to a record level in mid-April 2020. [11]. In addition, a decrease in the number of hospitalizations and the value of healthcare services provided was also observed [12]. So far, there are few publications in the medical literature on the functioning of non-uniform hospitals during the COVID-19 pandemic [13,14].

Ordinarily, the assessment of healthcare services has been based on statistical analyses using tables and classic charts [2,6,11,15,16,17]. The International Classification of Functioning, Disability and Health (ICF) can be used to assess health quality [18]. The ICF classification has been recognized by the World Health Organization as an international standard for describing health and health-related states [19]. In recent years, ICF has become present in the international arena [20,21]. The overall aim of the ICF is to provide a common operating language to facilitate data comparisons and create a systematic coding scheme for health information systems [22]. Moreover, this classification is used to monitor the functioning of health systems [23]. ICF-based documentation adds value in clinical practice [24], allowing smooth monitoring of the categories analyzed and the creation of “dynamic charts”, which are clear and simple to analyze for people without training in economics Moreover, it allows for a simple and clear analysis of several of the given categories simultaneously.

## 2. Aim of the Work

Assessment of the impact of a pandemic on the operation of a non-COVID-19 orthopedic and rehabilitation hospital using ICF categories.

## 3. Methodology

The study was retrospective in nature and included a global analysis of medical staff absences, the number of patients hospitalized, and the value of revenue earned from services provided in Q1 2020 compared with Q1 2019 at the Wiktor Dega Orthopedic and Rehabilitation Hospital in Poznań. The study was registered in the Clinical Trial Registry: NCT04521010, https://clinicaltrials.gov/ct2/show/NCT04521010 (accessed on 20 August 2020). This study was conducted in accordance with the Declaration of Helsinki. The study was approved by the Ethics Committee at the Karol Marcinkowski Memorial Medical University in Poznań (Approval No. 176/21 of 11 March 2021).

### 3.1. Statistical Analysis

Data were analyzed with Statistica software version 13.1. Descriptive statistics were reported as the mean and standard deviation (SD). The Shapiro–Wilk test was used to assess the normality of the distributions in the test scores. An independent Student’s t-test or Wilcoxon signed-rank tests were conducted to compare the differences between the results obtained in 2019 and 2020. A *p*-value of less than 0.05 was considered statistically significant.

### 3.2. ICF Classification

The results of the analysis of absences, the number of hospitalizations and the value of revenue generated in March and April 2019 and 2020 were evaluated depending on the mode of treatment (inpatient wards, day ward and specialist clinics) and the treatment profile (orthopedics, rehabilitation and rheumatology), and then recoded by categories and qualifiers of the ICF. The individual categories of the analyzed results were assigned appropriate codes according to the classification system.

Absence from work was coded in the category “d8502 full-time employment”. The following criteria were adopted: qualifier 0—if the employee worked from 96% to 100% of working time in a given month; qualifier 1—if the employee worked from 50% to 96% of working time in a given month; qualifier 2—if the employee worked from 25% to 49% of working time in a given month; qualifier 3—if the employee worked from 5% to 24% of working time in a given month; and qualifier 4—if the employee worked from 0% to 4% of working time in a given month.

The value of the revenue generated from services provided was coded in the category “d8701 public economic entitlements”. The following criteria were adopted: qualifier 0—if over 96% of a contract is fulfilled in a given month; qualifier 1—if between 50% and 95% of a contract is fulfilled in a given month; qualifier 2—if a between 25% and 49% of a contract is fulfilled in a given month; qualifier 3—if between 5% and 24% of a contract is fulfilled in a given month; and qualifier 4—if between 0% and 4% of a contract is fulfilled in a given month.

The percentage value of the number of hospitalized patients was coded in the category “e5800 health services”. The following criteria were adopted: qualifier 0—if 96% to 100% of patients were hospitalized in a given month; qualifier 1—if 50% to 95% of patients were hospitalized in a given month; qualifier 2—if 25% to 49% of patients were hospitalized in a given month; qualifier 3—if 5% to 24% of patients were hospitalized in a given month; and qualifier 4—if 0% to 4% of patients were hospitalized in a given month.

In the next stage, in order to better highlight the differences that occurred in the analyzed periods of time, the percentage distribution of ICF qualifiers in accordance with the classification designations is presented in graphic table form: qualifier 0—no problems: if the value of the percentage distribution was from 96 to 100%, a dark green color was assigned; 1—minor problems: if the value of the percentage distribution was from 50 to 95%, a light green color was assigned; qualifier 2—moderate problems: if the value of the percentage distribution was from 25 to 49%, a yellow color was assigned: qualifier 3—significant problems: if the value of the percentage distribution was from 5 to 24%, an orange color was assigned; and qualifier 4—extreme problems: if the value of the percentage distribution was from 0% to 4%, a red color was assigned.

## 4. Results

### 4.1. Statistical Analysis

In March 2020, compared to March 2019, the average working time of medical workers (*p* < 0.001) and the number of hospitalizations (*p* < 0.034) decreased significantly. In April 2020, compared to April 2019, the average working time of medical workers (<0.001) and the number of hospitalizations (0.002) also decreased significantly. In addition, in April 2020, the percentage value of the contracted services provided decreased significantly (*p* = 0.017), which was not observed in March of that year. In January and February 2020, there were no significant differences in any of the assessed categories. A detailed analysis is shown in Table 1.

### 4.2. ICF Classification

The presentation of the analysis based on ICF qualifiers (see Table 2) shows the scale of the severity of the problem and allows making appropriate decisions regarding corrective actions in the area of the treatment mode and profile.

Analyzing the percentage distribution of absences in the examined treatment types in March and April 2020, only about 50% of employees were present on all working days. In April, a significant increase in the percentage of employees absent from work for an entire month was observed. In the rehabilitation unit, a significant increase in absences above 25% was observed both in March and April.

In March, the contract terms were worked out in the in-patient orthopedics and rehabilitation department and in the orthopedics clinic. In April, the amount of contracted services was not fully provided in the in-patient wards: 21% on the orthopedics ward, 6% on the rehabilitation ward and 53% on the rheumatology ward. In the rehabilitation day ward, as early as in March, 47% of the value of contracted services were not provided, and in April, no services were provided. In the orthopedics outpatient clinic, 25% of services were not provided in March and 33% in April. Only the rheumatology outpatient clinic provided the full amount of contracted services in April.

In both March and April, there was a decrease in the number of patients admitted to the orthopedics, rehabilitation and rheumatology wards and specialist clinics. In March, no decrease in the number of hospitalizations was observed in the day ward, while in April it was 0%.

## 5. Discussion

The impact of the COVID-19 pandemic on private and professional life is being studied in many scientific fields. In our article, we have analyzed the impact of the pandemic on the operation of a non-COVID-19 hospital in terms of employee absence from work, the number of hospitalizations provided, and the income generated from healthcare services provided. The publications that have appeared to date have mainly evaluated COVID-19 hospitals [25].

In our study, the highest rate of absence of medical staff was in April (see Table 2). These results are consistent with surveys by Gaffney et al. [6], who used a mathematical analysis to present their results. It was found that the highest number of absences was also in this period, which coincided with the first peak in hospitalization and COVID-19 deaths, according to observations by Zheng et al. [11]. They used tables and a linear graph for analysis. As Alquezar et al. [2] have shown in their study of the impact of the coronavirus pandemic on the organization of Spanish hospital emergency departments, the degree of absence of hospital workers from work was not always related to the incidence of COVID-19. The authors presented the results of their study through statistical analysis presented in tables. In our article, we did not analyze the causes of absence from work, as this will be the subject of another article. On the basis of Chemali et al. [7], we can assume that the increased absences may have been affected by the stress, anxiety and intensified symptoms of occupational burnout. According to work by Dimitriu et al. [15], who also present their results only in the form of tables, the level of burnout found among medical personnel two months after the outbreak of the pandemic was higher than in studies conducted during normal working periods. It is worth citing the results of the study by Gaffney et al. [6], who stated that the publicity related to COVID-19 also caused workers with other symptoms than those of the infection to stay at home. Similar observations were made during the SARS pandemic in the early 21st century [16].

In the analyzed period, the value of the healthcare services provided and the number of hospitalized patients also decreased (see Table 1). As shown by Zagra et al. [26], due to the outbreak of the pandemic, there was a significant decrease in planned hospitalizations and surgical procedures, as well as consultations in outpatient clinics. They used a table and a bar chart in their analysis. Similar conclusions were published by Grass et al. [17], using tables in their results, and which were consistent with our observations. It is worth adding that these authors [18,19] examined orthopedic hospitals, while in our article we analyze orthopedic, rehabilitation and rheumatological treatment profiles (see Table 2). According to a report by the American Heart Association (AHA) [12], the revenues of hospitals and the healthcare system fell sharply as a result of the COVID-19 pandemic. This is consistent with our research, in which we found a significant decrease in the value of the revenue generated from services provided in April 2020 compared to April 2019.

The absence of employees and reduction in the number of patient hospitalizations (see Table 2) pose a threat to the maintenance of the hospital’s financial liquidity due to an inability to earn the contracted amount of revenue generated from the provision of healthcare services. Many patients, out of fear of contracting COVID-19, give up on or postpone medical care, which may further endanger their health [12]. Urgent action should be taken to support hospitals and healthcare systems.

The ICF classifications applied in this analysis help to assess the operation of a hospital in several categories simultaneously, and presenting the results in simple graphic form is a method that allows for a more legible, condensed and less complicated presentation of the problem under study in comparison to classical linear charts and tables used by the authors discussed above [2,6,7,11,15,16,17].

## 6. Conclusions

The COVID-19 pandemic has affected not only the operation of COVID-19 hospitals, but also of non-COVID-19 hospitals, causing an increase in staff absences, a decrease in the number of hospitalizations and in the value of earned income from the provision of healthcare services.

The ICF is a useful and simple tool for the evaluation of hospital healthcare services and simultaneous analysis of the studied areas.

## Figures and Tables

**Table 1:** Statistical analysis of the operation of the hospital in Q1 2019 and 2020.

ICF Category	Parameter Evaluated		Period											
			January 2019	January 2020	*p*	February 2019	February 2020	*p*	March 2019	March 2020	*p*	April 2019	April 2020	*p*
d 8502 Full-time employment	Working time (%)	Average ± SD	87.4 ± 20.9	87.6 ± 22.5	0.853	87.9 ± 22.1	88.5 ± 21.6	0.63	89.4 ± 21.1	81.8 ± 27.2	<0.001	88.1 ± 20.2	83.1 ± 30.8	<0.001
d 8701 Public economic entitlements	Value of the revenue from services provided (%)		93.5 ± 27.7	100.1 ± 33.0	0.478 *	100.4 ± 34.9	102.6 ± 29.7	0.51 *	101.6 ± 32.4	99.6 ± 45.5	0.688 *	106.6 ± 54.2	81.5 ± 41.8	0.017 *
e5800 Health services	Number of hospitalizations (n)		105.3 ± 73.4	102.1 ± 82.0	0.255 *	91.8 ± 77.8	93.3 ± 81.2	0.41 *	94.3 ± 74.3	87.6 ± 88.4	0.034 *	90.1 ± 77.8	48.0 ± 36.3	0.002 *

Dependent Student’s t-test; * Wilcoxon test.

**Table 2:** Percentage distribution of the evaluated International Classification of Functioning, Disability and Health (ICF) qualifiers by treatment profile.

**Orthopedics**							
**d8502 Full time employment**							
March 2020	3.40%	12%		8%	21.50%		55%
April 2020	7.50%	2.90%	5.40%	29.90%			54%
**d8701 Public economic entitlements**							
IN-PATIENT March 2020	127%						
IN-PATIENT April 2020	21%			79%			
CLINIC March 2020	109.81%						
CLINIC April 2020	47.60%						52.40%
**e5800 Health services**							
IN-PATIENT March 2020	8.20%	91.80%					
IN-PATIENT April 2020	17.30%			82.70%			
CLINIC March 2020	23.40%			76.60%			
CLINIC April 2020	33.30%				66.70%		
**Rehabilitation**							
**d8502 Full time employment**							
March 2020	4.10%	25%		11%	14.40%		45%
April 2020	11.90%		4.90%	12.60%	23.80%		47%
**d8701 Public economic entitlements**							
IN-PATIENT March 2020	110.48%						
IN-PATIENT April 2020	6%	94%					
DAY WARD March 2020	47.52%						52.48%
DAY WARD April 2020	0%						
**e5800 Health services**							
IN-PATIENT March 2020	3.60%	96.40%					
IN-PATIENT April 2020	33.30%				66.70%		
DAY WARD March 2020	115.70%						
DAY WARD April 2020	0%						
**Rheumatology**							
**d8502 Full time employment**							
March 2020	2.30%	9%	7%	25.60%	56%		
April 2020	11.40%		2%	31.80%		46%	
**d8701 Public economic entitlements**							
IN-PATIENT March 2020	14.46%		85.54%				
IN-PATIENT April 2020	53.27%						46.73%
CLINIC March 2020	22.89%			77.11%			
CLINIC April 2020	102.59%						
**e5800 Health services**							
IN-PATIENT March 2020	23.30%		76.70%				
IN-PATIENT April 2020	35.70%			64.30%			
CLINIC March 2020	37%				63%		
CLINIC April 2020	74.90%						25.10%

Red color—extreme problems; Orange color—significant problems; Yellow color—moderate problems; Light green color—minor problems; Dark green color—no problems.

## Data Availability

The data presented in this study are available on request from the corresponding author. The data are not publicly available due to ethical restrictions.
